# Inferring generation-interval distributions from contact-tracing data

**DOI:** 10.1098/rsif.2019.0719

**Published:** 2020-06-24

**Authors:** Sang Woo Park, David Champredon, Jonathan Dushoff

**Affiliations:** 1 Department of Ecology and Evolutionary Biology, Princeton University, Princeton, NJ, USA; 2 Department of Mathematics and Statistics, McMaster University, Hamilton, ON, Canada; 3 Department of Biology, McMaster University, Hamilton, ON, Canada; 4 Michael G. DeGroote Institute for Infectious Disease Research, McMaster University, Hamilton, ON, Canada; 5 Department of Pathology and Laboratory Medicine, University of Western Ontario, London, ON, Canada

**Keywords:** infectious disease modelling, generation interval, basic reproductive number, population structure, contact tracing

## Abstract

Generation intervals, defined as the time between when an individual is infected and when that individual infects another person, link two key quantities that describe an epidemic: the initial reproductive number, $R_{\mathrm{initial}}$, and the initial rate of exponential growth, *r*. Generation intervals can be measured through contact tracing by identifying who infected whom. We study how realized intervals differ from ‘intrinsic’ intervals that describe individual-level infectiousness and identify both spatial and temporal effects, including truncating (due to observation time), and the effects of susceptible depletion at various spatial scales. Early in an epidemic, we expect the variation in the realized generation intervals to be mainly driven by truncation and by the population structure near the source of disease spread; we predict that correcting realized intervals for the effect of temporal truncation but *not* for spatial effects will provide the initial forward generation-interval distribution, which is spatially informed and correctly links *r* and $R_{\mathrm{initial}}$. We develop and test statistical methods for temporal corrections of generation intervals, and confirm our prediction using individual-based simulations on an empirical network.

## 1. Introduction

An epidemic can be described by the exponential growth rate, *r*, and the reproductive number, R. The reproductive number is defined as the average number of secondary cases arising from a primary case; its value in a fully susceptible population, also known as the basic reproductive number $R_{0}$, is of particular interest as it provides information about the final size of an epidemic [1,2] as well as the endemicity level [3–5]. However, estimating the reproductive number directly from disease life history requires detailed knowledge, which is not often available, particularly early in an outbreak [6]. Instead, the reproductive number is often indirectly estimated from the exponential growth rate, which can be estimated from incidence data [7–11]. These two quantities are linked by generation-interval distributions [12–16].

At the individual level, a generation *interval* is defined as the time between when a person becomes infected and when that person infects another person [13]. While this definition is widely used in the literature, it is not directly related to a population-level *distribution*. There are important distinctions to be made when defining generation-interval distributions at the population level. The *intrinsic* generation-interval distribution describes the expected time distribution of infectious contacts made by a primary case [17]. On the other hand, *realized* generation-interval distributions describe the time between actual infection events over the course of an epidemic. Since some infectious contacts will be made with non-susceptible people, and thus not result in infection, realized distributions can differ systematically from the intrinsic distribution.

The shape of the realized generation-interval distribution depends on the reference time and perspective [17–21]. When an epidemic is growing exponentially, as often occurs near the beginning of an outbreak, the number of newly infected individuals will be large relative to the number infected earlier on. A susceptible individual is thus relatively more likely to be infected by a newly infected individual. Thus, ‘backward’ generation intervals, which look at a cohort of infectees and ask when their infectors were infected, will be shorter on average than intrinsic generation intervals—the converse is true when an epidemic is subsiding [17,19,21]. Likewise, we can define ‘forward’ generation intervals, which look at a cohort of infectors and ask when their infectees were infected. Mean forward generation intervals tend to decrease over the course of an epidemic as a result of susceptible depletion [17–20].

Realized generation intervals are also affected by spatial structure. In a population that does not mix homogeneously, susceptibility will tend to decrease more quickly in the neighbourhood of infected individuals than in the general population. This means that infectious contacts made late in an individual’s infection are more likely to be ineffective because of contacts that were made earlier (because the contactee may have been infected already). As a result, realized generation intervals (from the perspective of an infector) will typically have a shorter mean than the intrinsic generation-interval distribution in a non-homogeneous population. This perspective allows us to reinterpret the finding of [22] that, given an intrinsic generation interval and an observed growth rate, the reproductive number on various network structures is always smaller than would be predicted from homogeneous mixing.

In practice, realized generation intervals are often difficult to measure for many diseases, because it is difficult to observe when individuals become infected; in most cases, observable events are clinical (e.g. onset of symptoms). There are some exceptions: for example, generation intervals are commonly measured directly through contact tracing for canine rabies, where infection events are bites [23]. Intervals between observed disease progression events (commonly, onset of signs or symptoms) are called serial intervals [13]. Serial intervals are in many ways similar to generation intervals, but there are also complexities in their use [21]. We will not address these complexities here.

While an epidemic is ongoing, realized generation intervals, at least in theory, can be measured by identifying who infected whom and when, and aggregated to form a single distribution. We typically want to try to make inference based on this aggregated distribution — that is, on all available data that have been gathered since the beginning of an epidemic. These aggregated measurements are ‘truncated’ because we do not know what happens after the time of last observation. The distribution of these truncated intervals is similar to backward intervals during the exponential growth phase: there is a bias towards over-sampling shorter intervals, which are more likely to have concluded in time to be observed. We therefore predict that removing the truncation bias from aggregated generation intervals early in an epidemic will yield the initial forward generation-interval distribution, which contains information about the population structure and allows us to correctly infer the initial reproductive number from the initial exponential growth rate.

In this study, we explore spatio-temporal variation in realized generation intervals. We extend previous frameworks to investigate how aggregated generation-interval distributions change over time. We classify spatial effects on realized generation intervals into three levels (egocentric, local and global) and discuss how these affect realized generation-interval distributions. Finally, we compare two methods for accounting for temporal bias and test our prediction using individual-based simulations.

## 2. Intrinsic generation-interval distributions

**Figure 1.**
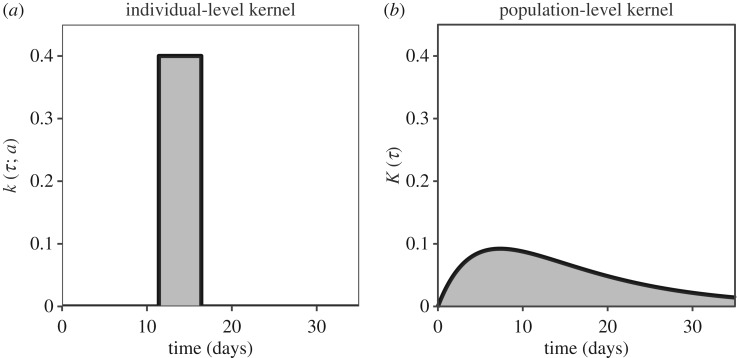
Comparison of individual- and population-level kernels. (*a*) An individual-level kernel of an infected individual with a latent period of 11.4 days followed by an infectious period of 5 days; this represents an individual realization of a random process. (*b*) A population-level kernel of infected individuals with latent and infectious periods exponentially distributed with means of 11.4 days and 5 days, respectively; this represents a population average of a random process. Shaded areas under the curves are equal to individual- and population-level reproductive numbers, both of which are set to 2 in this example. Parameters are chosen to reflect the West African Ebola outbreak [25].

Generation-interval distributions are often considered as population averages, but we can distinguish population-level distributions from individual-level distributions [13,24]; making this distinction clear will be particularly useful when we discuss spatial components later (figure 1). An individual-level intrinsic infection kernel *k*(*τ*; *a*) describes the rate at which an infected individual with ‘aspect’ *a* makes ‘infectious contacts’ (contacts which will cause infection if the contactee is susceptible). Individual aspects may represent variation in the course of infection (e.g. duration of latent and infectious periods) and the level of infectiousness, which can depend both on biological infectiousness and on contact patterns. Hereafter, we use *t*, *s* to represent calendar time and *τ*, *x* to represent time since infection.

Assuming that the individual properties are independent of risk of infection, the population-level kernel is given by integrating over these individual variations,

2.1 K(τ)=∫k(τ;a)f(a) da,

where *f*(*a*) represents a probability density over a (possibly multi-dimensional) aspect space. The population-level kernel describes the rate at which infectious contacts are made by an an infected individual, on *average*.

Assuming that a population mixes homogeneously, we can write

2.2 $$K(\tau )=R_{0}g(\tau ),$$

where $R_{0}=\int K(\tau )\;\text{d}\tau$ is the basic reproductive number (the expected number of secondary cases caused by a randomly chosen infectious individual in a fully susceptible population [1]) and *g*(*τ*) is the expected time distribution of infectious contacts made by a primary case (the intrinsic generation-interval distribution [17]). If the proportion of susceptibles contacted is not changing (e.g. in a homogeneously mixed population at the endemic equilibrium, or when the number of cases is vanishingly small), *g*(*τ*) also describes the realized (forward) generation intervals.

In a homogeneously mixing population, current disease incidence at time *t*, *i*(*t*), is the product of the current infectiousness of individuals infected in the past and the current proportion of the population susceptible, *S*(*t*),

2.3 $$i(t)=S(t)\int K(\tau )i(t-\tau )\;\text{d}\tau =R_{0}S(t)\int g(\tau )i(t-\tau )\;\text{d}\tau .$$

This model, referred to as the renewal equation, can describe a wide range of epidemic models [14,15,26–30]. Over a period of time where the susceptible proportion remains approximately constant (*S*(*t*) ≈ *S*(0)), we would expect approximately exponential growth in incidence *i*(*t*); assuming *i*(*t*) = *i*(0) exp(*rt*) yields the Euler–Lotka equation [31], which provides a direct link between the initial exponential growth rate *r* and the initial reproductive number $R_{\mathrm{initial}}=R_{0}S(0)$,

2.4 $$\frac{1}{R_{\mathrm{initial}}}=\int g(\tau )\exp(-r\tau )\;\text{d}\tau .$$

Under the homogeneous mixing assumption, the intrinsic generation-interval distribution *g*(*τ*) provides the correct link between *r* and $R_{\mathrm{initial}}$.

## 3. Realized generation-interval distributions across time

Realized generation intervals can be measured either forward (from the perspective of a cohort of infectors) or backward (from the perspective of a cohort of infectees) in time [17,21]. The forward generation-interval distribution *f*_*t*_(*τ*) describes the infection time of *infectees* caused by a cohort of infectors who were infected at time *t*. Similarly, the backward generation-interval distribution *b*_*t*_(*τ*) describes the infection time of *infectors* for a cohort of infectees who were infected at time *t*. For a single infector–infectee pair, both backward and forward *measurements* should give the identical generation interval. Therefore, the density of new infections occurring at time *t* + *τ* caused by individuals infected at time *t* can be expressed in terms of both the forward and backward generation-interval distributions,

3.1 $$R_{c}(t)i(t)f_{t}(\tau )=i(t+\tau )b_{t+\tau }(\tau ).$$

Here, $R_{c}(t)$ represents the case reproductive number, which is defined as the average number of secondary cases caused by a primary case infected at time *t* over the course of their infection [32].

In a homogeneously mixing population, the forward and backward generation-interval distributions can be calculated exactly. The density of new infections occurring at time *t* + *τ* caused by infectors who were infected at time *t* is given by

3.2 $$i_{t}(t+\tau )=R_{0}i(t)g(\tau )S(t+\tau ).$$

As shown in [17], the forward generation-interval distribution, *f*_*t*_(*τ*), is proportional to *i*_*t*_(*t* + *τ*),

3.3 $$f_{t}(\tau )=\frac{g(\tau )S(t+\tau )}{\int_{0}^{\infty }g(x)S(t+x)\;\text{d}x}.$$

In this case, the initial forward generation-interval distribution *f*_0_(*τ*) during the exponential growth phase (when *S*(*t*) ≈ *S*(0)) is equivalent to the intrinsic generation-interval distribution *g*(*τ*) and, therefore, provides the correct link between *r* and $R_{\mathrm{initial}}$. Likewise, the density of new infections occurring at time *t* caused by infectors who were infected at time *t* − *τ* is given by

3.4 $$i_{t-\tau }(t)=R_{0}i(t-\tau )g(\tau )S(t).$$

The backward generation-interval distribution, *b*_*t*_(*τ*), is proportional to *i*_*t*−*τ*_(*t*),

3.5 $$b_{t}(\tau )=\frac{i(t-\tau )g(\tau )}{\int_{0}^{\infty }i(t-x)g(x)\;\text{d}x}.$$

Substituting $R_{c}(t)=R_{0}\int_{0}^{\infty }g(\tau )S(t+\tau )\;\text{d}\tau$ confirms that equation (3.1) holds for this model.

During an ongoing epidemic, generation intervals cannot be measured for infection events that have not happened yet. This effect is called ‘right truncation’. Therefore, even if we aggregate all realized generation intervals by identifying who infected whom through contact tracing (assuming that infection events are observable), their mean will be shorter than the mean intrinsic generation interval. The aggregated generation-interval distribution, by definition, is a weighted average of backward generation-interval distributions (weighted by incidence) up until calendar time *t*,

3.6 $$a_{t}(\tau )\propto \int_{-\infty }^{t}i(s)b_{s}(\tau )\;\text{d}s.$$

The aggregated generation-interval distribution can also be expressed equivalently in terms of forward generation-interval distributions (weighted by incidence and case reproductive number),

3.7 $$a_{t}(\tau )\propto \int_{-\infty }^{t-\tau }R_{c}(s)i(s)f_{s}(\tau )\;\text{d}s.$$

Equation (3.1) confirms that both expressions are identical.

**Figure 2.**
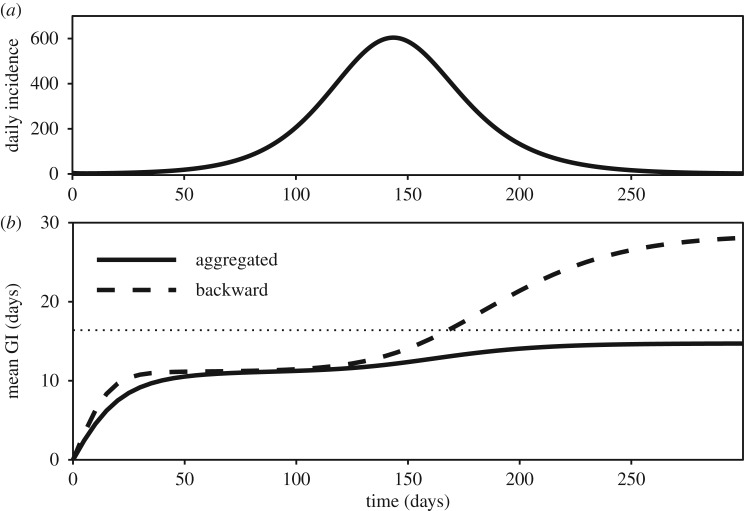
Temporal variation in the mean backward and aggregated generation interval. A deterministic susceptible–exposed–infectious–recovered (SEIR) model is simulated using Ebola-like parameters [25]: mean latent period 1/*σ* = 11.4 days, mean infectious period 1/*γ* = 5 days and the basic reproductive number $R_{0}=2$. The mean backward and aggregated generation intervals are calculated over the course of an epidemic. The dotted horizontal line represents the mean intrinsic generation interval.

For a single outbreak, the mean aggregated generation interval will always be shorter than the mean intrinsic generation interval (figure 2). There are two reasons for this phenomenon. First, longer generation intervals are more likely than short intervals to be missed because of right truncation. In particular, if we assume that the initial forward generation-interval distribution remains constant (*f*_*t*_(*τ*) ≈ *f*_0_(*τ*)) when an epidemic is growing exponentially (*i*(*t*) ≈ *i*(0) exp (*rt*) and $R_{c}(t)\approx R_{c}(0)$), the initial aggregated (or backward) generation-interval distribution is just the initial forward generation-interval distribution discounted by the rate of exponential growth [21],

3.8 $$a_{0}(\tau )=b_{0}(\tau )\propto f_{0}(\tau )\exp(-r\tau ).$$

A deterministic simulation confirms that the aggregated generation-interval distribution has the same mean as the backward generation-interval distribution during this period (figure 2). Second, the decreasing number of susceptibles over the course of an epidemic makes long infectious contacts less likely to result in infection [17]. Overall, we therefore expect naively using the aggregated generation-interval distribution to underestimate the initial reproductive number.

## 4. Realized generation-interval distributions across space

The effects of spatial structure on realized generation intervals can be understood in terms of effect of multiple contacts. Infected individuals may contact the same susceptible individual multiple times, but only the first infectious contact gives rise to infection in a given individual (after this, they are no longer susceptible). Therefore, we expect realized generation intervals from an individual in a spatially structured population to have a smaller mean than their mean intrinsic generation interval. To explore the effects of spatial structure on realized generation intervals, we relax our assumption that the population is homogeneous. Instead, we assume that a disease spreads on a network; infected individuals contact their ‘acquaintances’ at random, but ‘acquaintanceships’ are predetermined by the network structure before the beginning of an epidemic [22].

We first consider the infection process from an ‘egocentric’ point of view, taking into account infectious contacts made by a single infector. We define the egocentric kernel as the rate at which secondary infections are realized by a single primary case with aspect *a* in the absence of other infectors,

4.1 $$\hat{k}(\tau ;a)=k(\tau ;a)\exp\left(-\delta (a)\int_{0}^{\tau }k(x;a)\;\text{d}x\right),$$

where *k*(*τ*; *a*) is the individual-level intrinsic kernel and $e^{-\delta (a)\int_{0}^{\tau }k(x;a)\;dx}$ is the probability that a susceptible acquaintance has not yet been contacted by the focal individual. The dilution term, *δ*(*a*), models how contacts are distributed among the acquaintances.

Throughout this paper, we assume that there is a constant per-pair contact rate *λ* [22]. In this case, the intrinsic infectiousness of an individual R(a)=∫k(τ;a) dτ is the product of the number of acquaintances *N*(*a*), which can vary among individuals, the contact rate *λ* and the duration of infectious period; the dilution term is equal to the reciprocal of the number of acquaintances: *δ*(*a*) = 1/*N*(*a*). This assumption can be relaxed by allowing for asymmetry [22] or heterogeneity [33,34] in contact rates; for simplicity, we do not pursue these directions here.

The population-level egocentric kernel is found by integrating the individual-level kernel over individual variations,

4.2 $$\hat{K}(\tau )=\int \hat{k}(\tau ;a)f(a)\;\text{d}a,$$

where *f*(*a*) represents a probability density over a (possibly multi-dimensional) aspect space. Essentially, the population-level egocentric kernel accounts for the probability that a susceptible individual has not been infected by the focal individual. Trapman *et al.* [22] used this same kernel (also assuming a constant per-pair contact rate) to study the effect of network structure on the estimate of the basic reproductive number. The population-level egocentric generation-interval distribution is

4.3 $$\hat{g}(\tau )=\frac{\hat{K}(\tau )}{\int \hat{K}(x)\;\text{d}x}.$$

The population-level egocentric generation-interval distribution describes the distribution of times at which secondary infections are realized from an *average* infected–susceptible pair; for convenience, we will often omit ‘population level’. Finally, the initial exponential growth rate and the egocentric reproductive number are linked by the egocentric generation-interval distribution (and the Euler–Lotka equation) [22],

4.4 $$\frac{1}{R_{\mathrm{egocentric}}}=\int \hat{g}(\tau )\exp(-r\tau )\;\text{d}\tau .$$

As the egocentric distribution always has a shorter mean than the intrinsic distribution, $R_{\mathrm{egocentric}}$ will be smaller than $R_{\mathrm{initial}}$ estimated from the intrinsic distribution; this generation-interval-based argument provides an alternative biological interpretation for the result presented by [22].

For example, consider a susceptible–exposed–infected–recovered (SEIR) model, which assumes that latent and infectious periods are exponentially distributed. The intrinsic generation-interval distribution that corresponds to this model can be written as [30,35]

4.5 $$g(\tau )=\frac{\sigma \gamma }{\sigma -\gamma }(e^{-\gamma \tau }-e^{-\sigma \tau }),$$

where 1/*σ* and 1/*γ* are the mean latent and infectious periods, respectively. Assuming a constant per-pair contact rate of *λ* for any pair, we obtain the following egocentric generation-interval distribution:

4.6 $$\hat{g}(\tau )=\frac{\sigma (\gamma +\lambda )}{\sigma -(\gamma +\lambda )}(e^{-(\gamma +\lambda )\tau }-e^{-\sigma \tau }).$$

In this case, with constant transmission rate during the infectious period, the effect of accounting for pairwise contacts is the same as an increase in the recovery rate (by the amount of the per-pair contact rate *λ*). Infecting a susceptible contact is analogous to recovery because the contactee cannot be infected again—the infector can no longer transmit infection even if they are infectious (effectively losing infectiousness). Therefore, the resulting egocentric generation-interval distribution is equivalent to the intrinsic generation-interval distribution with mean latent period of 1/*σ* and mean infectious period of 1/(*γ* + *λ*). In practice, directly using the egocentric distribution to link *r* and $R_{\mathrm{initial}}$ using the Euler–Lotka equation is unrealistic because it requires that we know the per-pair contact rate. Instead, the per-pair contact rate can be inferred from the growth rate *r*, assuming that mean and variance of the degree distribution of a network is known (see [22] supplementary material, §1.4.2); we briefly describe this relationship in §7.3.

**Figure 3.**
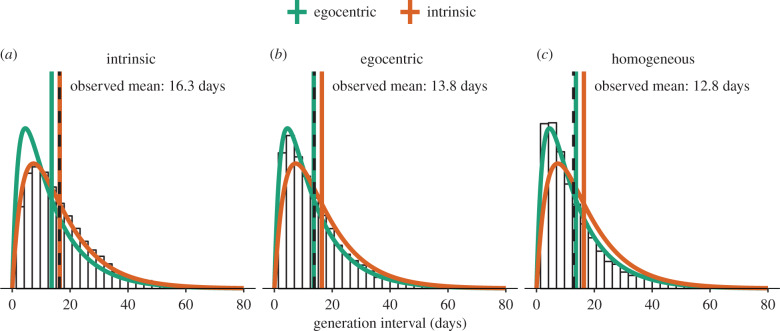
Spatial effects on realized generation intervals. Theoretical distributions and means are shown in colour (and are the same in each panel, for reference). Simulated distributions and means are shown in black. (*a*) The intrinsic generation-interval distribution corresponds to all infectious contacts by a focal individual, regardless of whether the contact results in infection. (*b*) The egocentric generation-interval distribution corresponds to the distribution of all infectious contacts by the focal individual with susceptible individuals, in the case where the focal individual is the only possible infector (simulated on a star network). (*c*) Realized generation-interval distributions have a shorter mean than egocentric distributions in general, because contacts can be wasted when susceptibles become infected through other routes (simulated on a homogeneous network). All figures were generated using 5000 stochastic simulations on a network with five nodes (one infector and four susceptibles) with Ebola-like parameters [25]: mean latent period 1/*σ* = 11.4 days and mean infectious period 1/*γ* = 5 days. Per-pair contact rate *λ* = 0.25 days^−1^ is chosen to be sufficiently high so that the differences between generation-interval distributions are clear. Each simulation is run until all individuals are either susceptible or have recovered.

This calculation can be validated by simulating stochastic infection processes on a ‘star’ network (i.e. a single infected individual at the centre connected to multiple susceptible individuals who are not connected with each other). Simulations (figure 3) confirm that in this case the distribution of *contact* times matches the intrinsic generation-interval distribution (*a*), while the distribution of realized generation intervals (i.e. *infection* times) matches the egocentric generation-interval distribution (*b*).

The egocentric generation interval (equation (4.3)) only explains some of the reduction in realized generation intervals that occurs on most networks, however. Generation intervals are also shortened by indirect connections: a susceptible individual can be infected through another route before the focal individual makes infectious contacts. Simulations on a small homogeneous network (i.e. complete network) confirm this additional effect (figure 3*c*). We can think of simulations on this network as an approximation of (local) infection process in a small household, consisting of five individuals; we expect realistic local network structures (and their effects on the realized generation intervals) to lie between a star network and a complete network.

In general, spatial reduction in the mean realized generation interval can be viewed as an effect of susceptible depletion and can be further classified into three levels: egocentric, local and global. Egocentric depletion, as discussed previously, is caused by an infected individual making multiple contacts to the same individual. Local depletion refers to a depletion of susceptible individuals in a household or neighbourhood; we can think of these structures as small homogeneous networks embedded in a larger population structure (and therefore we can expect similar effects to those seen in figure 3*c*). Both the egocentric and local depletion effects can be observed early in an epidemic, especially in a highly structured population, even if most of the population remains susceptible. Finally, global depletion refers to overall depletion of susceptibility at the population level, and explains the reduction in realized compared with intrinsic generation intervals that occurs even in a well-mixed population (figure 2).

## 5. Inferring the initial forward generation-interval distribution

In a large homogeneously mixing population, the initial forward generation-interval distribution is equivalent to the intrinsic distribution and provides the correct link between the exponential growth rate *r* and the initial reproductive number $R_{\mathrm{initial}}$ (see equation (3.3)). In a non-homogeneous population, the initial forward generation-interval distributions are subject to spatial effects and, therefore, are different from the intrinsic distribution. Since spatial effects have the same effect on how the epidemic spreads as they do on realized generation intervals, we expect the initial forward generation-interval distributions, which implicitly account for the spatial structure, to correctly link *r* and $R_{\mathrm{initial}}$ through the Euler–Lotka equation (equation (2.4)). Spatial effects on realized generation intervals are generally expected to be analytically intractable, even in simple networks (e.g. see [20] for discussion regarding the realized generation intervals in a household with one infector and two susceptibles); therefore, we rely on simulations to validate this prediction.

**Figure 4.**
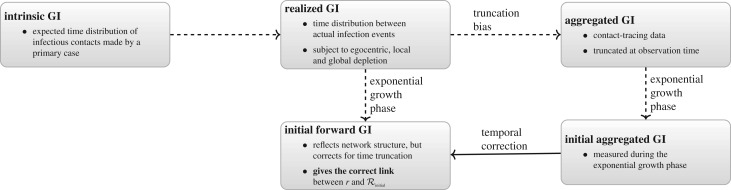
A summary of spatiotemporal effects on generation intervals. The intrinsic generation-interval distribution describes the expected time distribution of infectious contacts made by a primary case. The realized (forward) generation-interval distribution describes the time between actual infection events—egocentric, local and global depletion of the susceptible pool reduces the mean realized generation intervals. The aggregated generation-interval distribution reflects the contact-tracing process during an ongoing epidemic and is subject to right truncation bias. Our goal is to estimate the initial forward generation-interval distribution from the initial aggregated generation-interval distribution, measured during the exponential growth period.

When realized generation intervals are aggregated over the course of an epidemic, there will be four effects present in the data (figure 4): (i) right-truncation effect, (ii) egocentric depletion effect, (iii) local depletion effect, and (iv) global depletion effect. We can correct explicitly for the egocentric effect and, in the case of exponential growth, the right-truncation effect; these effects shorten the mean realized generation intervals, which in turn will reduce the estimate of the reproductive number [15,16]. While the other two effects are difficult to measure, we can make qualitative predictions about their effects on the realized generation intervals and reproductive numbers: both local and global depletion effects also reduce the number of infections that occur and shorten generation intervals. If we can correct for the truncation bias early in an outbreak, during the exponential growth phase, we should be able to infer the initial forward generation-interval distribution, which incorporates egocentric and local spatial effects but not the global effects, from the aggregated distribution.

Here, we investigate two methods for correcting for temporal bias in aggregated generation-interval data (see Methods for details). We refer to the first method as the population-level method as it relies on realized generation intervals aggregated across the entire population. When an epidemic is growing exponentially, right truncation causes the aggregated generation interval to be discounted by the exponential growth rate (equation (3.8)); hence, we can ‘undo’ the truncation by exponentially weighting the aggregated generation-interval distribution [19–21],

5.1 $$f_{0}(\tau )\propto a_{0}(\tau )\exp(r\tau ),$$

where *r* is the exponential growth rate.

We refer to the second method as the individual-level method because it relies on individual contact information. We model each infection as a non-homogeneous Poisson process arising from the infector (equation (7.11)); incorporating information about time of infection of an infector, time of infection of an infectee and time since the beginning of an epidemic allows us to explicitly model the truncation process in the realized generation intervals. For both methods, the mean and coefficient of variation (CV) of the initial forward generation-interval distributions are estimated by maximum likelihood; the inferred generation-interval distributions are then used to estimate the initial reproductive number $R_{\mathrm{initial}}$ from the observed growth rate *r* using the Euler–Lotka equation.

**Figure 5.**
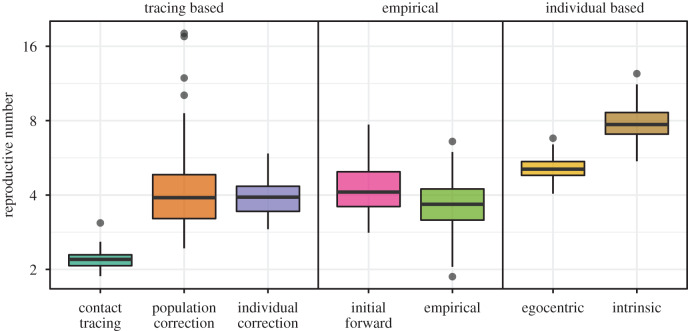
Comparison of estimates of reproductive number based on various methods. Using the aggregated generation-interval distributions (based on the first 1000 realized generation intervals) without correcting for right-truncation (labelled as ‘contact tracing’) severely underestimates the reproductive number. Similarly, using the intrinsic generation-interval distribution overestimates the reproductive number because it fails to account for local spatial effects; the egocentric distribution corrects for this only partially. Both population-level and individual-level methods provide estimates of reproductive number that are consistent with the empirical estimates, which we define as the average number of secondary cases generated by the first 75 infected individuals, as well as the estimates based on the initial forward generation intervals, which are calculated by applying the Euler-Lotka equation to the realized generation intervals of all infections caused by the first 75 infected individuals. Boxplots are generated using 100 stochastic simulations of the SEIR model on an empirical network using Ebola-like parameters [25]: mean latent period 1/*σ* = 11.4 days and mean infectious period 1/*γ* = 5 days. Per-pair contact rate *λ* = 0.08 days^−1^ is chosen to be sufficiently high such that differences are clear.

To test these methods, we simulate 100 epidemics with Ebola-like parameters on an empirical network [36] and compare the estimates of the initial reproductive number with empirical reproductive numbers, which we define as the average number of secondary cases generated by the first 75 infected individuals, as well as the initial reproductive number calculated from the empirical initial forward generation intervals, which we define as the generation intervals for all infections caused by the first 75 infected individuals (figure 5). For simplicity, we assume that realized generation intervals are observed without error and assume that there is no under-reporting of generation intervals. We do not expect under-reporting to affect the inference of generation-interval distributions (see electronic supplementary material, appendix A.3) unless there are systematic biases in the observation process. On the other hand, it is difficult to measure generation intervals precisely because (i) infection events are often unobserved and (ii) there may be multiple potential infectors; these factors can introduce biases to the estimates of the initial reproductive number [21]. We do not pursue these directions in this study.

As expected, estimating the reproductive number based on the intrinsic generation-interval distribution overestimates the empirical reproductive number; estimates based on the egocentric generation-interval distribution (equation (4.3)) address this problem only partially, as they do not account for indirect (local) spatial effects. Direct estimates based on the aggregated generation intervals from contact tracing (via Euler–Lotka) severely underestimate the empirical estimates. While both population- and individual-level corrections provide similar estimates to the empirical estimates (as well as to estimates based on the untruncated empirical initial forward generation-interval distribution) on average, population-level estimates are more variable as they are more sensitive to outliers in generation intervals and our estimates of the initial exponential growth rate. For smaller values of $R_{\mathrm{initial}}$, we expect the differences to become smaller. In the electronic supplementary material, we present the same figure using smaller $R_{\mathrm{initial}}$ (see electronic supplementary material, appendix A.1) and using Erlang-distributed latent periods (see electronic supplementary material, appendix A.2), which better corresponds to Ebola. Overall, our qualitative conclusions do not change.

## 6. Discussion

The intrinsic generation-interval distribution, which describes the expected time distribution of infectious contacts, provides a direct link between speed (initial exponential growth rate, *r*) and strength (initial reproductive number, $R_{\mathrm{initial}}$) of an epidemic in a homogeneously mixing population [13,15,16,24]. However, realized generation-interval distributions can vary depending on how and when they are measured [17,19–21]; determining which distribution correctly links *r* and $R_{\mathrm{initial}}$ can be challenging. Here, we analyse how realized generation intervals aggregated over the course of an epidemic, possibly through contact tracing, differ from intrinsic generation intervals. Changes due to right truncation reflect observation bias, whereas changes due to spatial or network structure reflect the dynamics of the outbreak. Thus, correcting the aggregated distribution for temporal, but not spatial, effects provides the correct link between *r* and $R_{\mathrm{initial}}$.

Realized generation intervals that have been aggregated over the course of an epidemic are subject to right truncation—it is not possible to trace individuals who have not been infected yet. The aggregated distributions can be thought of as averages of ‘backward’ generation intervals (measured by looking at infectors of a cohort of individuals infected at the same time) [17–21]. During an ongoing outbreak, the aggregated generation-interval distribution will always have a shorter mean than the intrinsic-interval distribution because of right truncation. Early in the outbreak, the initial aggregated intervals are expected to match the initial backward intervals. Near the end of an outbreak, the effect of right truncation becomes negligible but the aggregated generation intervals are still shorter on average than intrinsic generation intervals, because of depletion of the susceptible population.

We think of susceptible depletion as operating on three levels: egocentric, local and global. Egocentric susceptible depletion refers to the effect of an infected individual making multiple contacts to the same susceptible individual. Accounting for the egocentric effect allows us to link the results by [22] to established results based on generation intervals. Local susceptible depletion refers to the effect of multiple ‘linked’ individuals (e.g. in the same household or neighbourhood) making infectious contacts to the same susceptible individual. Global susceptible depletion refers to the decrease in the susceptible proportion of the whole population.

Susceptible depletion happening at all three levels shortens realized generation intervals but acts on different time scales. Egocentric and local depletion effects are present from the beginning of an epidemic, even when depletion in the global susceptible population is negligible and can strongly affect the initial spread of an epidemic. Therefore, we predict the realized generation intervals during an exponential growth phase to contain information about the contact structure, allowing us to estimate the initial forward generation-interval distribution by simply accounting for the right truncation. Simulation studies confirm our prediction: using the initial forward generation-interval distribution provides the correct link between *r* and $R_{\mathrm{initial}}$.

We compare two methods for estimating the initial forward generation-interval distribution and assume that the initial forward generation-interval distribution follows a gamma distribution. The gamma approximation of the generation-interval distribution has been widely used because of its simplicity [9,37–40]; we previously showed that a gamma approximation (requiring estimation of only two parameters) can be sufficient to understand the role of generation-interval distributions in linking *r* and $R_{\mathrm{initial}}$ for Ebola, rabies and measles [16]. However, further investigation of our methods suggests that making a wrong distributional assumption can lead to biased estimates of the mean and CV of a generation-interval distribution (see electronic supplementary material, appendix A.4), even though the estimated gamma distribution may ‘look’ indistinguishable from the true shape of the intrinsic generation-interval distribution (derived from the SEIR model). These results are particularly alarming because it is impossible to know the true shape of the generation-interval distribution for real diseases. Nonetheless, biases in the parameter estimates of a generation-interval distribution may have opposite effects on the estimate of $R_{\mathrm{initial}}$ (e.g. shorter mean generation interval leads to lower $R_{\mathrm{initial}}$ whereas narrower generation-interval distribution leads to higher $R_{\mathrm{initial}}$) and, therefore, may have small effects on the overall estimate of $R_{\mathrm{initial}}$ (see electronic supplementary material, appendix A.4).

Generation-interval-based approaches to estimating the reproductive number often assume that an epidemic grows exponentially [12,14–16]. In practice, heterogeneity in population structure can lead to subexponential growth [41–46]; we therefore expect our simulations on an empirical network to be better characterized by subexponential growth models [46]. However, our simulations suggest that the initial exponential growth assumption still provides a viable approach for estimating the reproductive number.

Contact tracing provides an effective way of collecting epidemiological data and controlling an outbreak [47–49]. In particular, using tracing information allows us to infer real-time estimates of the time-varying reproductive number [50–53]. Generation-interval distributions, which can be either assumed or estimated, often play a central role in analysing tracing data. Our study illustrates that realized generation intervals over the course of an epidemic contain information about the underlying contact structure, which can be implicitly reflected in the estimates of the reproductive number; this perspective can be particularly useful for characterizing an epidemic because detailed information about the contact structure is often unavailable.

The generation-interval distribution is a key, and often under-appreciated, component of disease modelling and forecasting. Different definitions, and different measurement approaches, produce different estimates of these distributions. We have shown that estimates based on aggregated generation intervals (e.g. measured through contact tracing) differ in predictable ways from intrinsic estimates based on underlying measures of infectiousness (e.g. from shedding studies). These predictable differences can arise from temporal effects, egocentric spatial effects, local spatial (or network) effects and population-level effects. Correcting aggregated intervals for temporal effects allows us to estimate a spatially informed initial forward distribution, which accurately describes how disease spreads in a population. Future studies should carefully consider how measurement influences estimated generation-interval distributions, and how these distributions influence the spread of disease.

## 7. Methods

### 7.1. Deterministic SEIR model

To study the effects of right truncation on the realized generation intervals, we use the deterministic SEIR model. The SEIR model describes how disease spreads in a homogeneously mixing population; it assumes that infected individuals become infectious after a latent period. We use a SE^*m*^I^*n*^R model, which extends the SEIR model to have multiple equivalent stages in the latent and infectious periods. This gives latent and infectious periods with Erlang distributions (gamma distributions with integer shape parameters, including the exponential distribution), which are often more realistic than the exponentially distributed periods in the standard SEIR model [54,55],

7.1 $$\begin{array}{rl} \frac{\text{d}S}{\text{d}t} & =-\beta S\sum_{k=1}^{n_{I}}I_{k},\\ \frac{\text{d}E_{1}}{\text{d}t} & =\beta S\sum_{k=1}^{n_{I}}I_{k}-n_{E}\sigma E_{1},\\ \frac{\text{d}E_{m}}{\text{d}t} & =n_{E}\sigma (E_{m-1}-E_{m})\;\text{for }m=2,3,\ldots ,n_{E},\\ \frac{\text{d}I_{1}}{\text{d}t} & =n_{E}\sigma E_{m}-n_{I}\gamma I_{1}\\ \;\text{and}\;\;\frac{\text{d}I_{n}}{\text{d}t} & =n_{I}\gamma (I_{n-1}-I_{n})\;\text{for }n=2,3,\ldots ,n_{I}, \end{array}\}$$

where *S* is the proportion of susceptible individuals, *E*_*m*_ is the proportion of exposed individuals in the *m*-th compartment and *I*_*n*_ is the proportion of infectious individuals in the *n*-th compartment. Parameters of the model are specified as follows: *β* is the transmission rate, 1/*σ* is the mean latent period, *n*_*E*_ is the number of latent compartments, 1/*γ* is the mean infectious period and *n*_*I*_ is the number of infectious compartments. We scale the proportions of individuals in each compartment by the total population size *N*. In the main text, we present results based on exponentially distributed latent and infectious periods; we show results based on Erlang distributed latent periods (*n*_*E*_ = 2), which better match the incubation period distribution of Ebola virus disease (see electronic supplementary material).

### 7.2. Stochastic SEIR model

We simulate an individual-based SEIR model on a contact network, using an algorithm based on the Gillespie algorithm [56,57]. We begin by randomly selecting individuals assumed to be infected at *t* = 0. For each infected individual *i*, we randomly draw the latent period *E*_*i*_ from an Erlang distribution with mean 1/*σ* and shape *n*_*E*_. We then construct the random infectious period and infectious contact times simultaneously as follows. For each of the *n*_*I*_ stages of the infectious period, we draw the number of infectious contacts (before transitioning to the next compartment) from a geometric distribution with probability *n*_*I*_*γ*/(*S*_*i*_*λ* + *n*_*I*_*γ*), where *S*_*i*_ is the number of susceptible acquaintances and *λ* is the per-pair contact rate. We then choose the time between consecutive events (the chosen number of contacts, followed by exit from the given stage of infection) from an exponential distribution with rate *S*_*i*_*λ* + *n*_*I*_*γ*. For each contact, a contactee is uniformly sampled from the set of susceptible acquaintances of the individual *i*. The infectious period *I*_*i*_ is the sum of all of these waiting times.

After repeating the contact process for all initially infected individuals, all contacts are put into a sorted queue. The first person in the queue becomes infected (thus decreasing *S*_*i*_ by 1 for all individuals *i* that are acquaintances of the newly infected individual), and the current time is updated to infection time of this individual. Any subsequent contacts made to this individual are removed from the queue because they will no longer be effective. We repeat the contact process for this newly infected individual. Then, new contacts are added to the sorted queue. The simulation continues until there are no more contacts left in the queue.

### 7.3. Egocentric relationship between *r* and R (SEIR model)

Here, we show that the egocentric relationship between *r* and R derived by Trapman *et al.* [22] (see the original source for detailed derivations) matches what would be calculated by applying the Euler–Lotka equation to the egocentric (rather than the intrinsic) generation-interval distribution. Assume that latent and infectious periods are exponentially distributed with mean 1/*σ* and 1/*γ*, respectively. Assuming a constant per-pair contact rate of *λ* for any pair, the egocentric generation-interval distribution can be written

7.2 $$\hat{g}(\tau )=\frac{\sigma (\gamma +\lambda )}{\sigma -(\gamma +\lambda )}(e^{-(\gamma +\lambda )\tau }-e^{-\sigma \tau }).$$

Substituting into equation (4.4), we get

7.3 $$R_{\mathrm{egocentric}}=\left(1+\frac{r}{\sigma }\right)\left(1+\frac{r}{\gamma +\lambda }\right),$$

where *r* is the exponential growth rate. Alternatively, the egocentric reproductive number can be expressed based on the degree distribution (mean *μ* and variance *v*) of a network,

7.4 $$R_{\mathrm{egocentric}}=\underset{\mathrm{average}\;\mathrm{contact}\;\mathrm{rate}}{\underbrace{\kappa \lambda }}\times \underset{\mathrm{mean}\;\mathrm{effective}\;\mathrm{infectious}\;\mathrm{period}}{\underbrace{\frac{1}{\gamma +\lambda }}},$$

where *κ* = *v*/*μ* + *μ* − 1, referred to as the mean degree excess [58], describes the expected number of susceptible individuals that an average infected individual will encounter early in an outbreak. Combining the two equations, we get

7.5 $$\lambda =\frac{\left(\gamma +r)(\sigma +r\right)}{(\kappa -1)\sigma -r},$$

which completes the relationship between the growth rate and the egocentric reproductive number [22],

7.6 $$R_{\mathrm{egocentric}}=\frac{\gamma +r}{\gamma \sigma /(\sigma +r)+r/\kappa }.$$

### 7.4. Estimating the initial forward generation-interval distribution

The *population-level method* estimates the initial forward generation-interval distribution by reversing the inverse exponential weighting in the aggregated generation-interval distribution without explicitly accounting for the infection process (i.e. who infected whom) [19–21],

7.7 $$f_{0}(\tau )\propto a_{0}(\tau )\exp(r\tau ),$$

where *r* is the initial exponential growth rate. In order to do so, we first approximate the aggregated distribution *a*_0_ with a gamma distribution by assuming that realized generation intervals (subject to right truncation) during the exponential growth phase come from the same gamma distribution; specifically, we estimate the mean $\bar{G}$ and shape *α* of a gamma distribution by maximum likelihood. Then, the initial forward generation-interval distribution follows a gamma distribution with mean $\alpha /(\alpha /\bar{G}-r)$ and shape *α*. We then use the estimated initial forward generation-interval distribution to infer the initial reproductive number $R_{\mathrm{initial}}$ from the estimated growth rate (using the Euler–Lotka equation).

The *individual-level method* models each infection *i* from an infected individual *j* as a non-homogeneous Poisson process between the time at which infector *j* was infected (*t*_*j*_) and the truncation time (*t*_truncate_), with time-varying Poisson rate at time *t* equal to *Λf*_0_(*t* − *t*_*j*_), where *f*_0_(*t*) is the initial forward generation-interval distribution [59]. We use a gamma distribution (parameterized by its mean and shape) to model the initial forward generation-interval distribution. Then, the probability that an individual *j* infects *n*_*j*_ individuals between *t*_*j*_ and *t*_truncate_ is equal to

7.8 $$\frac{\Lambda^{n_{j}}F_{0}{\left(t_{\mathrm{truncate}}-t_{j};\theta \right)}^{n_{j}}\exp(-\Lambda \;F_{0}(t_{\mathrm{truncate}}-t_{j};\theta ))}{n_{j}!},$$

where *θ* is a (vector) parameter of the initial forward generation-interval distribution *f*_0_ (and the corresponding cumulative distribution function *F*_0_). On the other hand, the probability density that the realized generation interval between infector *j* and infectee *i* is equal to 0 ≤ *s*_*i*,*j*_ ≤ *t*_truncate_ − *t*_*j*_ can be expressed using a truncated distribution [60,61],

7.9 $$\frac{\;f_{0}(s_{i,j};\theta )}{F_{0}(t_{\mathrm{truncate}}-t_{j};\theta )}.$$

Therefore, the probability density that individual *j* infects *n*_*j*_ individuals between *t*_*j*_ and *t*_truncate_ with realized generation intervals *s*_*i*,*j*_ for $i=1,\ldots ,n_{j}$ is a product of equation (7.8) and equation (7.9),

7.10 $$\frac{\Lambda^{n_{j}}\exp(-\Lambda \;F_{0}(t_{\mathrm{truncate}}-t_{j};\theta ))\prod_{i=1}^{n_{j}}f_{0}(s_{i,j};\theta )}{n_{j}!}.$$

The full likelihood of contact-tracing data, which include aggregated generation intervals as well as information about who infected whom, until time *t*_truncate_ can be written as

7.11 $$\begin{array}{rl} & L(\Lambda ,\theta \;|\;s,t,n,t_{\mathrm{truncate}})\\ & \;=\prod_{\;j=1}^{N_{I}}\left(\frac{\Lambda^{n_{j}}\exp(-\Lambda \;F_{0}(t_{\mathrm{truncate}}-t_{j};\theta ))\prod_{i=1}^{n_{j}}f_{0}(s_{i,j};\theta )}{n_{j}!}\right), \end{array}$$

where *N*_*I*_ is the total number of infected individuals (in the data). This likelihood is a special case of the likelihood suggested in [62], which assumes that the second event (observation of infection) occurs simultaneously with the corresponding initiating event (infection of an individual). Here, we estimate parameters *Λ* and *θ* by maximum likelihood. In theory, the forward generation intervals arising from the same infector may be correlated because of non-independence in the contact process [35]; although we do not account for this potential correlation in our likelihood, our simulations (figure 5) suggest that approximating the initial forward generation-interval distribution with a single distribution provides a viable approach for estimating the reproductive number.

We use the estimated distribution *f*_0_ to infer the initial reproductive number $R_{\mathrm{initial}}$ from the estimated growth rate (using the Euler–Lotka equation). When the entire transmission process is known, we expect *Λ* to match $R_{\mathrm{initial}}$; otherwise, *Λ* will be sensitive to under-reporting of the number of infections caused by each infected individual. As we show in electronic supplementary material, appendix A.3, the estimates of $R_{\mathrm{initial}}$ using the Euler–Lotka equation from *f*_0_ remain unbiased even in the presence of random under-reporting.

### 7.5. Measuring the exponential growth rate

We estimate the initial exponential growth rate *r* of an epidemic from daily incidence by modelling the cumulative incidence *c*(*t*) with a logistic function [11],

7.12 $$c(t)=\frac{K}{1+[(K/c_{0})-1]\;e^{-rt}}.$$

While the exponential growth rate (i.e. the rate of change in log incidence) of the logistic function changes throughout an epidemic, we focus strictly on estimating the *initial* exponential growth rate (when *t* → −∞). The method of estimating the initial exponential growth rate by fitting a logistic curve has been previously validated against simulations of stochastic compartmental models [11].

Fitting directly to cumulative incidence can lead to overly confident results [63]; instead, we fit interval incidence *x*(*t*) = *c*(*t* + Δ*t*) − *c*(*t*), where Δ*t* is 1 day, to daily incidence, assuming that daily incidence follows a negative binomial distribution with overdispersion parameter *θ*. We estimate parameters *r*, *K*, *c*_0_ and *θ* by maximum likelihood. The fitting time window is defined from the last trough before the peak of an epidemic to the first day after the peak of an epidemic.

### 7.6. Empirical network

To simulate epidemics on a realistic network, we use the ‘condensed matter physics’ network from the Stanford Large Network Dataset Collection [36]. This graph describes co-authorship among anyone who submitted a paper to the Condensed Matter category in arXiv between January 1993 and April 2003 [64]. It consists of 23 133 nodes and 93 497 edges. The same network was used by [22] to study how network structure affects the estimate of the basic reproductive number.

## Supplementary Material

Appendix

## Data accessibility

All code is available at https://github.com/parksw3/contact_trace.
