# Supplementary material for: Inferring generation-interval distributions from contact-tracing data
Source: J R Soc Interface. 2020 Jun 24;17(167):20190719. doi: 10.1098/rsif.2019.0719 (PMC7328397; doi:10.1098/rsif.2019.0719)
Supplement: Appendix [file rsif20190719supp1.pdf]

A Appendix

A.1 Comparison of estimates of reproductive number – smaller $\mathcal{R}$

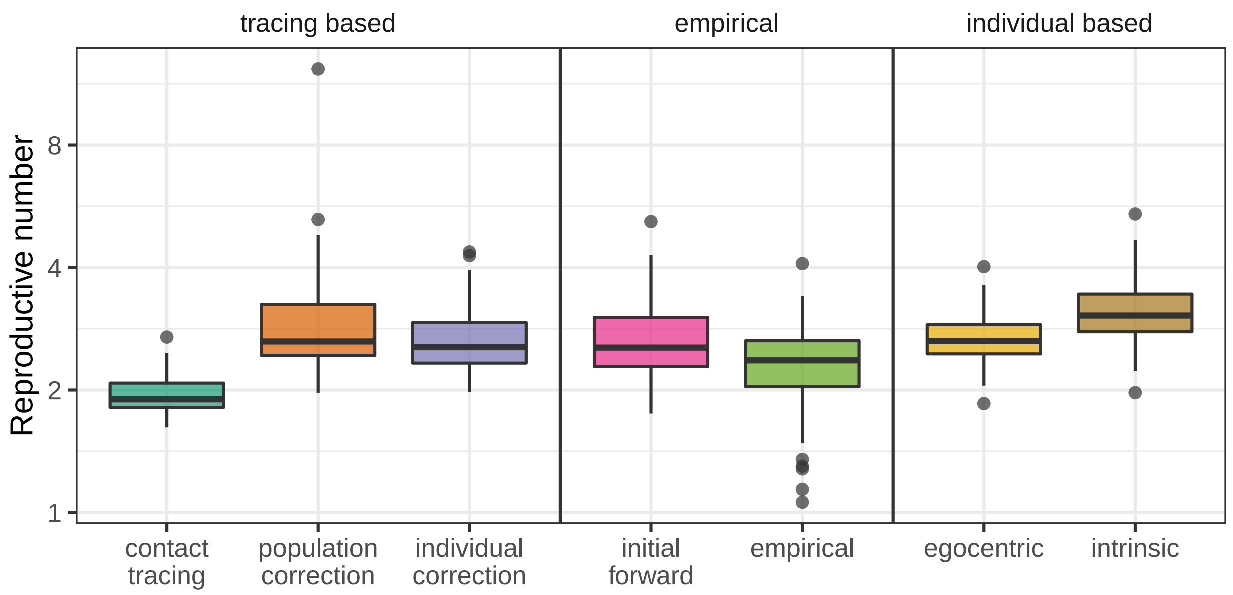

Figure A.1: **Comparison of estimates of reproductive number based on various methods.** This figure matches Fig. 5 in the main text, but with a smaller per-pair contact rate ($\lambda = 0.026$ days^{-1}) used to simulate epidemics. All other parameters are the same as in Fig. 5.

A.2 Comparison of estimates of reproductive number – Erlang distributed latent periods

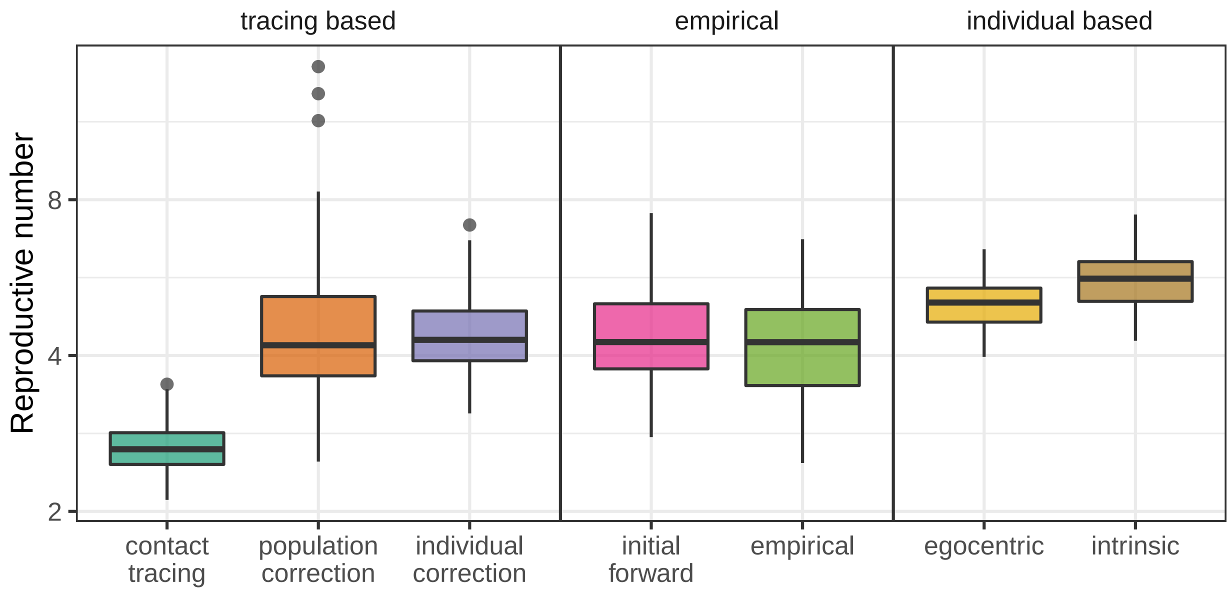

Figure A.2: **Comparison of estimates of reproductive number based on various methods.** This figure matches Fig. 5 in the main text, but an Erlang-distributed latent period ($n_E = 2$ instead of 1) is used to simulate epidemics; this assumption better matches the incubation period distribution of the Ebola virus disease [1]. All other parameters are the same as in Fig. 5 in the main text.

A.3 Comparison of estimates of reproductive number – under-reporting of generation intervals

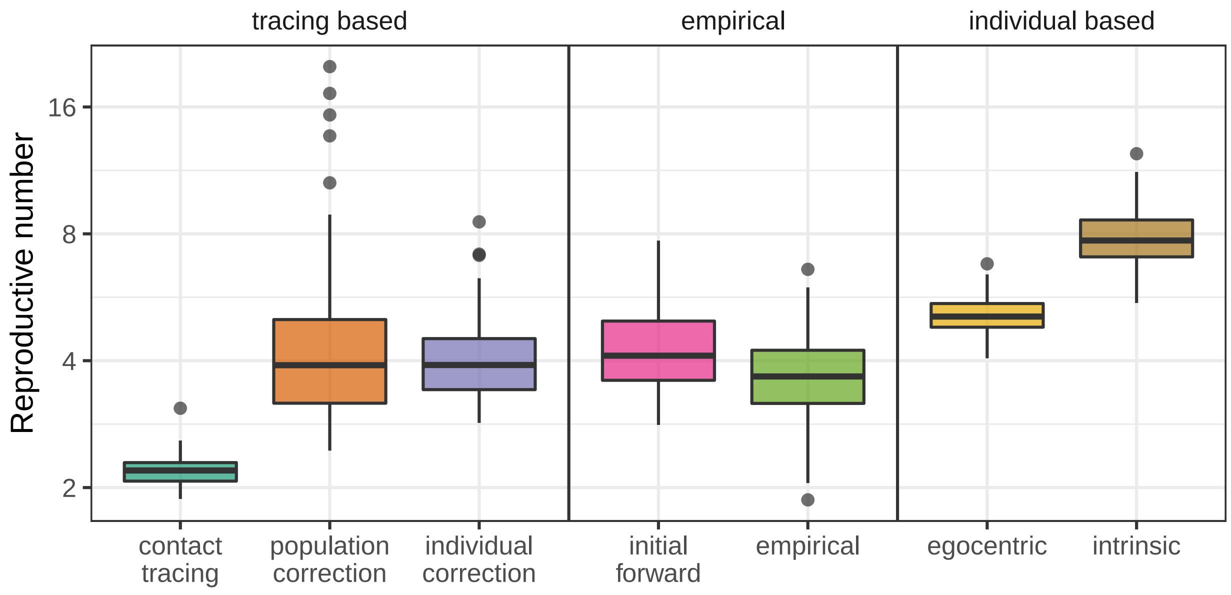

Figure A.3: **Comparison of estimates of reproductive number based on various methods.** This figure matches Fig. 5 in the main text, except that we assume that not all generation intervals are observed. Instead, we assume that each generation interval independently has a 30% probability of being reported when we apply population- and individual-based methods to estimate $\mathcal{R}_{\text{initial}}$. Independence is a simplifying assumption that should not affect the conclusions. We still obtain unbiased estimates of $\mathcal{R}_{\text{initial}}$ when generation intervals are under-reported. All parameters (and boxplots not based on these methods) are the same as in Fig. 5 in the main text.

A.4 Testing the individual-based method on simulations on a homogeneous network

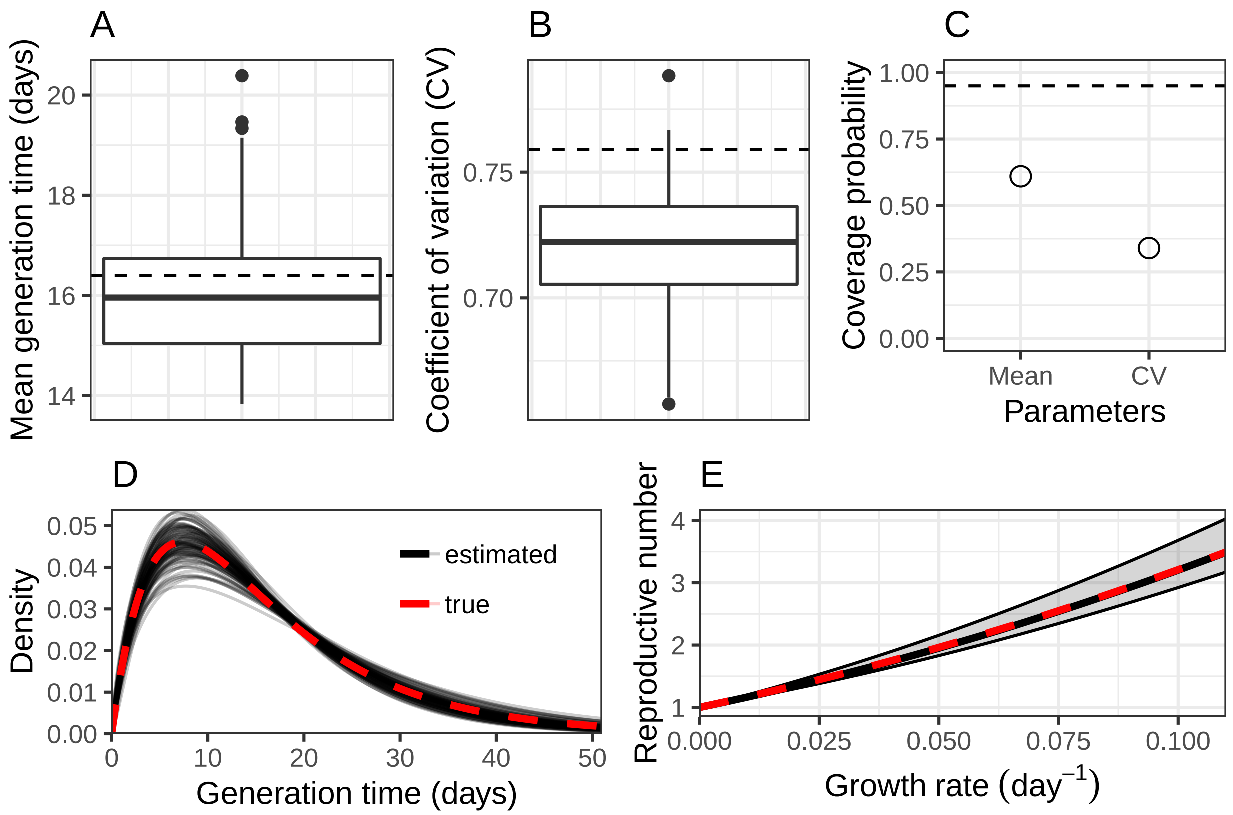

Figure A.4: **Wrong distributional assumptions may result in biased estimates of the parameters of a generation-interval distribution.** We simulate a stochastic SEIR model on a homogenous network with 10^5 individuals using Ebola-like parameters [1]: mean latent period $1/\sigma = 11.4$ days, mean infectious period $1/\gamma = 5$ days, and the basic reproductive number $\mathcal{R}_0 = 2$. Then, we apply the individual-based method based on the first 1000 infections. (A-B) A boxplot of 100 estimates of the mean generation intervals and their coefficient of variations (CV). Dashed horizontal lines represent the true value. (C) Coverage probability of the mean generation intervals and their CVs based on the 95% confidence interval. Dashed horizontal lines represent the 95% coverage probability. (D) Estimated generation-interval distributions based on the gamma approximation and the true generation-interval distribution of an SEIR model. (E) Estimated r–$\mathcal{R}$ relationships based on the gamma approximation and a true r–$\mathcal{R}$ relationship of an SEIR model. Even though there are biases in the estimates of the parameters of the generation-interval distribution, the r–$\mathcal{R}$ relationship is much less biased: a shorter mean generation interval decreases $\mathcal{R}$ whereas a tighter generation-interval distribution increases $\mathcal{R}$ [2; 3].

References

[1] WHO Ebola Response Team . 2014 Ebola virus disease in West Africa–the first 9 months of the epidemic and forward projections. *N. Engl. J. Med.* **371**, 1481–1495.

[2] Wallinga J and Lipsitch M. 2007 How generation intervals shape the relationship between growth rates and reproductive numbers. *Proc. R. Soc. B.* **274**, 599–604.

[3] Park SW, Champredon D, Weitz JS, and Dushoff J. 2019 A practical generation-interval-based approach to inferring the strength of epidemics from their speed. *Epidemics.* **27**, 12–18.
